# Altered Spontaneous Brain Activity in Children with Early Tourette Syndrome: a Resting-state fMRI Study

**DOI:** 10.1038/s41598-017-04148-z

**Published:** 2017-07-06

**Authors:** Yue Liu, Jieqiong Wang, Jishui Zhang, Hongwei Wen, Yue Zhang, Huiying Kang, Xu Wang, Wenfeng Li, Huiguang He, Yun Peng

**Affiliations:** 1 Department of Radiology, Beijing Children’s Hospital, Capital Medical University, National Center for Children’s Health, Beijing, China; 2 State Key Laboratory of Management and Control for Complex Systems, Institute of Automation, Chinese Academy of Sciences, Beijing, China; 3 University of Chinese Academy of Sciences, Beijing, China; 4 Department of Neurology, Beijing Children’s Hospital, Capital Medical University, National Center for Children’s Health, Beijing, China; 5 Center for Excellence in Brain Science and Intelligence Technology, Chinese Academy of Sciences, Beijing, China

## Abstract

Tourette syndrome (TS) is a childhood-onset chronic disorder characterized by the presence of multiple motor and vocal tics. This study investigated the alterations of spontaneous brain activities in children with TS by resting-state functional magnetic resonance imaging (rs-fMRI). We obtained rs-fMRI scans from 21 drug-naïve and pure TS children and 29 demographically matched healthy children. The amplitude of low-frequency fluctuation (ALFF), fractional ALFF (fALFF) and regional homogeneity (ReHo) of rs-fMRI data were calculated to measure spontaneous brain activity. We found significant alterations of ALFF or fALFF in vision-related structures including the calcarine sulcus, the cuneus, the fusiform gyrus, and the left insula in TS children. Decreased ReHo was found in the right cerebellum. Further analysis showed that the ReHo value of the right cerebellum was positively correlated with TS duration. Our study provides empirical evidence for abnormal spontaneous neuronal activity in TS patients, which may implicate the neurophysiological mechanism in TS children. Moreover, the right cerebellum can be potentially used as a biomarker for the pathophysiology of early TS in children.

## Introduction

Tourette syndrome (TS) is a developmental neuropsychiatric disorder characterized by chronic motor and vocal tics, which begins at the age of 6 to 7 years and lasts for more than one year^1^. This is a common disorder with prevalence rates ranging from 0.05% to 3%, which could also cause impairments including distress, social impact, interference with activities, etc.^2^. Practically, TS is diagnosed according to Diagnostic and Statistical Manual of Mental Disorders IV (DSM-IV) that focuses on patients’ behavior and the history of tics. However, the DSM-IV pays little attention to brain abnormalities of patients, and these brain abnormalities have been proved by a bunch of neuropathological studies^3^.

As is well known, an aberrant distribution of interneurons in the cortico–striato–thalamo–cortical (CSTC) circuit was found in TS patients^4–7^. Previous studies with non-invasive magnetic resonance imaging (MRI) found a trend towards reduced volumes of the lenticular nuclei (putamen and globus pallidus combined) in TS boys^8^. Grey matter volumes in the right inferior frontal gyrus and the left frontal pole were reduced in TS patients without associated comorbidities relative to healthy controls^9^. TS patients also show a decrease of the white matter volume in the right frontal pole as well as significantly increased axial diffusivity and mean diffusivity in the right cingulum bundle projecting to the cingulate gyrus. More importantly, these structural changes were found to be significantly correlated with tic severity and duration^10, 11^. They also found a decrease of fractional anisotropy and an increase of radial diffusivity in the deep white matter tracts of the CSTC circuit and superficial white matter of the primary motor and somatosensory cortex, commissural and association fibers^12^. These studies reveal the importance of studying brain alterations in TS patients.

Besides previous structural studies of TS, the changes of neural activity in TS patients have also been studied in recent studies. Neural activity is a sensitive measurement that has been observed to be acutely altered by brain structural lesions^13^. Functional magnetic resonance imaging (fMRI) is a widely used imaging technique that indirectly tracks neural activity via a blood oxygenation level dependent (BOLD) contrast signal during different cognitive and behavioral tasks. Peterson’s study found that TS adults showed a decreased putamen and the frontal cortical activity during tic suppression^14^. In start-cue analysis of the brain activity, the affected regions of the brain were bilateral frontal regions, temporo-parietal regions, the precuneus, and the thalamus in a group of older children and adolescents with TS^15^. However, it is difficult to make TS children perform specified task to collect task-related fMRI data.

Resting-state functional MR imaging (rs-fMRI) has been found to be a powerful tool for evaluating spontaneous neural activity^16–18^ of participants who do not perform a certain task. Rs-fMRI has been widely used in clinical research, especially in children^19, 20^. Amplitude of low frequency fluctuations (ALFF)^21^, fractional ALFF (fALFF)^22^, and regional homogeneity (ReHo)^23^ obtained from the rs-fMRI data are the three commonest indices used to quantify the neural activity. ALFF represents the intensity of low-frequency oscillations (LFO), and fALFF represents the relative contribution of specific LFO to the whole detectable frequency range^24^. ALFF is more reliable than fALFF in gray matter regions, whereas fALFF is more specific than ALFF in that fALFF can effectively suppress artifacts in non-specific brain regions, such as the ventricles and the vicinity of blood vessels^22, 24^. ReHo measures the neural synchronization of a given voxel with its neighboring voxels^23^. A previous study indicated that ReHo was more sensitive than ALFF for detecting regional abnormalities and that ALFF may be complementary to ReHo for measuring global spontaneous activity^25^. Therefore, the combination of these three methods may provide more information about the pathophysiological framework in the human brain than either method alone^25^.

So far, only one study combined ALFF and fALFF to investigate the abnormal spontaneous brain activity in TS patients^26^. In the present study, we not only investigated abnormal intensity of neural activity via ALFF/fALFF analysis, but also investigated abnormal neural synchronization via ReHo analysis in TS children. We hypothesized that 1) significant differences of ALFF/fALFF and ReHo values would be detected within specific brain regions between normal controls and TS children; and 2) the alterations of the spontaneous brain activity would be related to tic severity scores or tic duration in TS children.

## Materials and Methods

### Subjects and data acquisition

The study enrolled a total of 75 participants including 33 TS patients and 42 normal controls by Beijing Children’s hospital, Beijing, China. All the enrolled patients met DSM-IV-TR criteria for TS. We used a clinical interview and the Children’s Yale-Brown Obsessive Compulsive Scale (CY-BOCS)^27^ to diagnose obsessive compulsive disorder (OCD) and the German short version of Wender Utah rating scale (WURS-k, translated to Chinese)^28^ to diagnose attention deficit hyperactivity disorder (ADHD). All 33 TS patients were without OCD. Tic severity for all patients was rated using the Yale Global Tic Severity Scale (YGTSS)^29^ and ranged from 10 to 79 (mean ± SD: 46.50 ± 18.037). The duration of TS ranged from 3 month to 5 years (mean ± SD: 1.81 ± 1.423 years). For those who had a course less than 1 year, TS diagnosis was made by follow-up call. This study was approved by the Medical Ethics Committee of Beijing Children’s Hospital, Beijing, China. All subjects signed the informed consent after they were explained the whole study. And the study was carried out in accordance with relevant guidelines by the Medical Ethics Committee of Beijing Children’s Hospital, including MR scan and clinical diagnosis and treatment. Eight patients with concurrent ADHD were excluded in this study.

A Philips 3 T scanner was applied to scan all participants to acquire resting-state fMRI images and T1-weighted images. The scanner parameters for fMRI data are TR/TE = 2000/24 ms, slice thickness = 3 mm, matrix = 64 × 64, field of view (FOV) = 22 × 22 cm^2^. The scanner parameters for T1-weighteed images are TR/TE = 8.19/3.78 ms, slice thickness = 1 mm, matrix = 256 × 256, FOV = 20 × 20 cm^2^. All patients have been recorded TS duration and measured disease severity by a Chinese translation of the YGTSS.

### Image preprocessing

The standard preprocessing of the resting-state fMRI images was preprocessed by the toolbox DPARSF (V2.3, http://www.restfmri.net/forum/DPARSF)^30^. The images in the first 10 time points of each time series were removed to allow for subjects’ adaption to the scanning and the magnetization equilibration. Then, the fMRI volumes of the remaining time points were slice corrected to the middle slice of each volume. In order to reduce the effects of head motion, we adopted the following steps: 1) The 3 translational and 3 rotational motion parameters were computed. The fMRI data were excluded from further analysis if the head movement over 2 mm translation or 2° angular rotation in any axis; 2) The framewise displacement (FD) was calculated. The data were also excluded if the mean FD of the subject exceeded 0.3 mm; 3) The Friston 24-parameter model including six head motion parameters, six head motion parameters one time point before, and the 12 corresponding squared items^31^, was used to regress out head motion effects in the preprocessing (individual-level correction) as recommended in the previous paper^32^; 4) the mean FDs were considered as confounding variables in both the group-level comparison and the correlation of “brain indices – clinical parameters”^32, 33^. After subject exclusion, 21 TS patients and 29 normal controls were chosen for the study. The nuisance covariate effects of white matter signal and CSF signal were also removed by a linear regression process. After that, the regressed data were spatial normalized to the Montreal Neurological Institute (MNI) template and resampled to 3 × 3 × 3 mm cubic voxels.

### Measurement of ALFF/fALFF and ReHo

To calculate ALFF, we firstly performed the spatial smoothing on the resampled images with a 4 mm full width at half maximum (FWHM) Gaussian kernel. Then we converted the smoothed signal of each voxel from time domain to frequency domain via Fast Fourier Transform (FFT) to obtain the power spectrum. This power spectrum (frequency range: 0–0.25 Hz) was square-rooted at each frequency, and then averaged across 0.01–0.08 Hz at each voxel, which was taken as ALFF^21^. To calculate fALFF, we divided the sum of the amplitude (square root of power spectrum) across 0.01–0.08 Hz was divided by that of the entire frequency range (0–0.25 Hz)^22^. Finally, all the ALFF/fALFF maps were divided by the mean value of each ALFF/fALFF map.

To measure ReHo, the band-pass filtering (0.01–0.08 Hz) on the normalized images was performed. ReHo was quantified by the Kendall coefficient between a voxel and its neighbors^23^. Then ReHo value of each voxel was divided by the mean value of the ReHo map. Finally, smoothing was done with a 4 mm FWHM Gaussian kernel for the results.

### Statistical analysis and correlation analysis

In the statistical analysis, group comparisons of demographic data and head motion between TS patients and normal controls were conducted using two-sample t test and χ^2^ test in SPSS (release 17.0). A two-sample t test with the age, sex, intracranial volume (ICV) and mean FD as confounding variables was performed on the maps of ALFF, fALFF, ReHo to obtain functional differences between TS patients and normal controls (cluster-wise FDR corrected, p < 0.001)^34, 35^, respectively. The significant clusters were labelled by the coordinate of the peak voxel.

In order to investigate the relationship between the brain abnormality and the clinical parameters (TS duration and YGTSS), we firstly calculated the average value of ALFF within the clusters with significant ALFF changes obtained by the two sample t test, respectively. Then Pearson’s correlation coefficients between the averaged ALFF value and clinical parameters were calculated in the patient group, considering the effect of age, sex, ICV and mean FD. Similar to ALFF, the correlation analysis was performed on fALFF and ReHo, respectively. The multiple correlations were corrected by Bonferroni correction.

## Results

### Demographic and clinical characteristic

Table 1 shows the general clinical information of the TS patients and normal controls. No significant difference was found between normal controls and TS patients in sex (*p* = 0.33) or age (*p* = 0.11). All subjects used in this study were without ADHD or OCD. No significant differences were found in mean FD (*p = *0.55) between the two groups.

**Table 1:** Demographic variables and clinical characteristics of TS patients and normal controls.

Characteristics	TS patients (n = 21)	Normal controls (n = 29)	p-value
Sex	16 M/5 F	19 M/11 F	0.33^†^
Age	8.7 ± 3.0	10.1 ± 3.1	0.11^*^
YGTSS	44.6 ± 17.9	—	—
Duration (months)	17.9 ± 14.4	—	—
Head motion (mean FD)	0.14 ± 0.04	0.13 ± 0.06	0.55^*^

YGTSS = Yale Global Tic Severity Scale; FD = framewise displacement; M = male; F = female. ^*^Two-sample t test. ^†^χ^2^ test.

### Altered ALFF/fALFF and ReHo in TS patients

**Figure 1:**
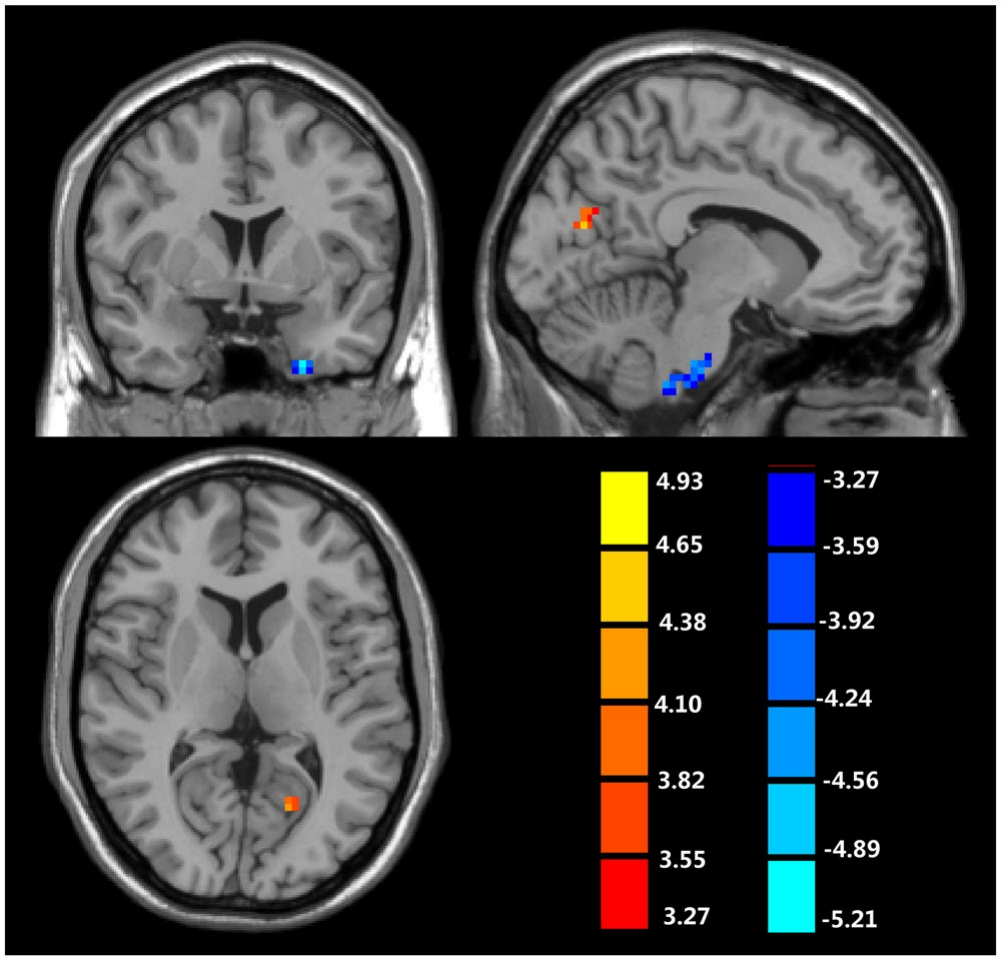
The regions with abnormal ALFF in patients with Tourette syndrome when comparing with normal controls (cluster-wise FDR corrected, p < 0.001). Cold represents decreased ALFF while hot represents increased ALFF.

Table 2 and Fig. 1 show an increased ALFF in the left calcarine sulcus, the left cuneus of TS patients when compared to the normal controls. Significantly decreased ALFF was found in the left cerebellum and the left fusiform gyrus.

**Table 2:** The regions with abnormal ALFF in patients with Tourette syndrome when comparing with normal controls (cluster-wise FDR corrected, p < 0.001).

Type	Anatomical location	Hemisphere	x	y	z	Peak *T*-value	Cluster size (voxels)
Controls > TS	Cerebellum	Left	−12	−27	−42	−5.26	36
	Fusiform gyrus	Left	−24	3	−42	−5.37	34
Controls < TS	Calcarine sulcus	Left	−24	−69	15	4.68	49
	Cuneus	Left	−9	−75	18	4.26	24

x, y, z: the coordinate in MNI space; TS: Tourette syndrome.

**Figure 2:**
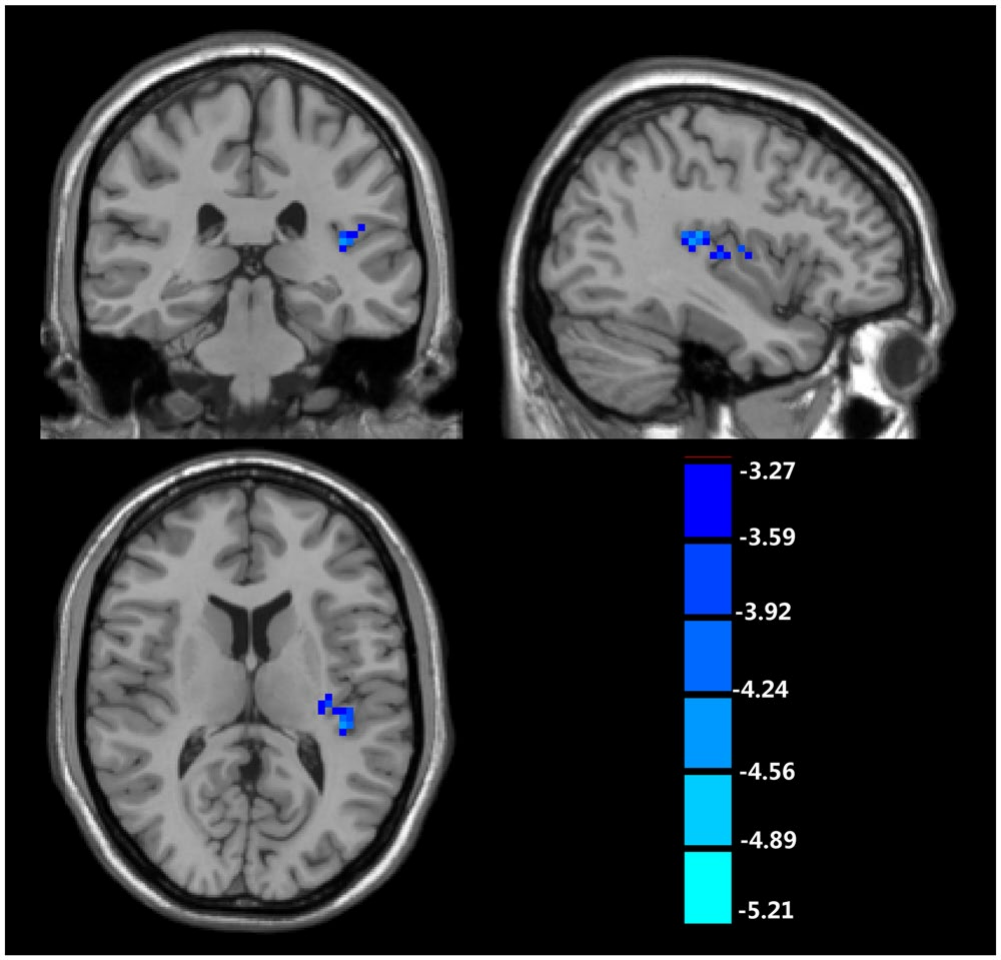
The regions with decreased fALFF in patients with Tourette syndrome when comparing with normal controls (cluster-wise FDR corrected, p < 0.001).

Table 3 and Fig. 2 show that decreased fALFF was found in the left insular cortex of TS patients. No significantly increased fALFF was found in TS patients.

**Table 3:** The regions with abnormal fALFF in patients with Tourette syndrome when comparing with normal controls (cluster-wise FDR corrected, p < 0.001).

Type	Anatomical location	Hemisphere	x	y	z	Peak *T*-value	Cluster size (voxels)
Controls > TS	Insula	Left	−39	−30	12	−4.86	47

x, y, z: the coordinate in MNI space (cluster maxima); TS: Tourette syndrome.

**Figure 3:**
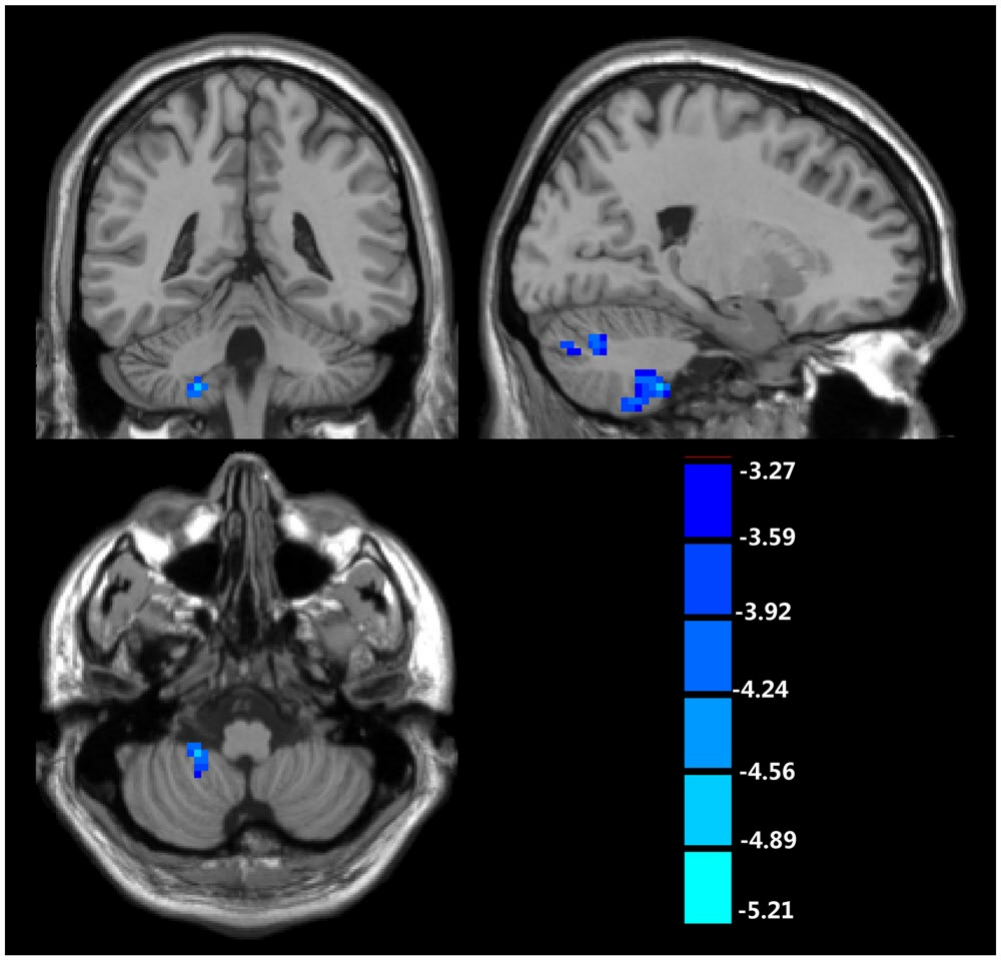
The regions with decreased ReHo in patients with Tourette syndrome when comparing with normal controls (cluster-wise FDR corrected, p < 0.001).

As shown in Fig. 3 and Table 4, the ReHo values were significantly decreased in the right cerebellum of TS patients while no significantly increased values were observed in TS patients.

**Table 4:** The regions with abnormal ReHo in patients with Tourette syndrome when comparing with normal controls (cluster-wise FDR corrected, p < 0.001).

Type	Anatomical location	Hemisphere	x	y	z	Peak T-value	Cluster size (voxels)
Controls > TS	Cerebellum	Right	24	−66	−36	−4.98	94
			21	−42	−51	−4.68	47

x, y, z: the coordinate in MNI space. TS: Tourette syndrome.

### Correlation with clinical parameters

**Figure 4:**
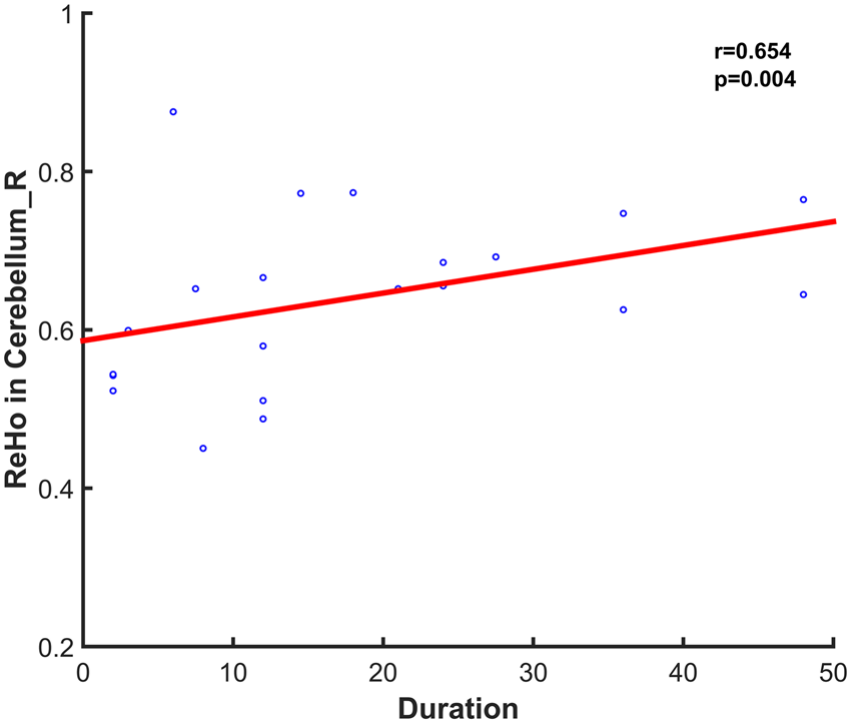
Positive correlations between the ReHo of abnormal clusters and TS duration in patients with Tourette syndrome, controlling for the effect of age, gender, ICV, and mean FD.

After calculating the correlation coefficients between the brain abnormalities of TS patients and clinical parameters (YGTSS and TS duration), significantly positive correlation was only observed between the ReHo of the cerebellum and TS duration (*r = *0.654, *p = *0.004) in TS patients (Fig. 4). No significant correlation was found between the other brain indices and YGTSS, TS duration.

## Discussion

ALFF/fALFF and ReHo analyses have been used to investigate the intrinsic neuropathology of various mental disorders^25, 36–38^. These three methods are based on different neurophysiological mechanisms, wherein ALFF/ fALFF analysis demonstrates neural intensity and ReHo demonstrates neural coherence. In this study, abnormal neural activity was detected by three methods in several brain regions. The combination of three methods can reveal more comprehensive functional changes than those reflected by a single method. Only one study investigated the abnormal spontaneous brain activity in TS patients, but it is with a small sample size of 17 TS cases^26^. Our study has a larger sample size with 21 TS cases, which will lead to better reliability. Therefore, we believe our results represent reliable information that is necessary for understanding the abnormalities of neural activity in TS patients.

When compared with healthy controls, TS children showed a significant increase of ALFF in the left calcarine sulcus and the left cuneus as well as a significant decrease in the left cerebellum and the left fusiform gyrus. FALFF and ReHo were decreased in the left insula and the right cerebellum, respectively. These results showed that the changes of spontaneous brain activities were mostly located in the vision-related structures, the insula and the cerebellum. We further found that ReHo of the right cerebellum was positively correlated with TS duration.

### Abnormal neural activity in vision-related structures

The increased intensity of the neural activity was mainly located at the vision-related structures (left calcarine sulcus, the left cuneus and left fusiform gyrus). Using a different type of analysis such as volume analysis method, Peterson and colleagues^39^ found larger volume in parieto-occipital cortex and smaller volume in the inferior occipital cortex of young children with TS. And regional cerebral volumes were significantly associated with the severity of tic symptoms in parieto-occipital regions. The temporo-occipital association cortex was related to complex perceptual function of language and vision in the patients^10^. In order to maintain control over tics as well as eye blinks, humming or clearing the throat and so on, the special control system was more constantly active in patients with TS versus control subjects. The abnormal activity of the control system aimed to be suited to additional demand, such as from a directed task. Therefore, abnormal connections between the occipital lobule and the temporal regions may underlie premonitory sensory urges preceding tics in TS. Our result that TS children with greater neural activity in the vision-related structures suggests that the reorganization of vision-related structures in TS is either modulated to compensate for or resulted from tic-related movements^40^.

### Disruption in insula

Our study revealed the decrease of fALFF in the left insular cortex. Another study found changes in the dorsal anterior cingulate, bilateral insula/frontal operculum, and frontal and parietal regions prior to tic onset in TS^41^. Our study is consistent with their results. The insular cortex is a part of the cingulo-opercular network. The cingulo-opercular network is responsible for the set-maintenance and makes brain resistant to distraction^42^. The abnormalities of this network may affect task-maintenance processes resulting in unwanted breakthroughs (i.e. tics) of normally suppressed behaviors in TS patients^42^. Moreover, the insula is also a part of the widespread network and is tightly connected with cortical and subcortical areas. It has reciprocal connections with the primary motor cortex as well as multiple connections with the limbic system including amygdala, claustrum, and thalamic nuclei^43–46^. The connection between the insula and the primary motor cortex and that between the insula and the limbic system perform complex integrative functions related to the organization and initiation of movement. Thus, the insula may be regarded as potentially modifying relay points in tic generation. In a word, the decreased neural activity in the insula suggests the decreased control function, leading to tics in TS children.

### Disruption in cerebellum

Our study showed decreased ReHo values in the right cerebellum. In humans, the cerebellum plays an important role in motor control, and it may also be involved in some cognitive functions such as attention and language as well as in regulating fear and pleasure responses^47^. Functional imaging studies have shown cerebellar activation in relation to language, attention, and mental imagery; correlation studies have shown interactions between the cerebellum and non-motor areas of the cerebral cortex; and a variety of non-motor symptoms have been recognized in people with damage that appears to be confined to the cerebellum^48, 49^. Few studies have investigated the role of the cerebellum in TS^50^. The cerebellum takes part in two cortico-cerebellar networks in verbal working memory, language development in children. Scott *et al*. found that the right cerebellar lesions impaired language development in children^51^. Previous studies pointed out that the cerebellum exhibited activation one second before the onset of tics^52^. Furthermore, Tobe and colleagues^50^ found that the TS group aged from 6 to 60 years showed gray matter reductions in the lateral cerebellar hemispheres that appeared to correlate with tic severity. Disruption in the cerebellum may be related with the phenomena that patients involuntarily talk dirty. Thus, the cerebellum plays a significant role in TS^50^.

More importantly, we found that the ReHo of the right cerebellum were positively correlated the tic duration, which suggests neural abnormalities in language cortex related to tics duration. This change in TS children can be interpreted as signs of neural plasticity in response to the experiential demand. The results of our analysis are consistent with the TS patients’ symptoms of tics on the one hand, and also point out the important role of the language cortex in the pathophysiologic pattern of early TS children on the other hand.

### Discrepancies between ALFF and fALFF

We would like to emphasize that the fALFF is defined as the ALFF divided by the total power in the entire detectable frequency range (0–0.25 Hz). The differential findings between ALFF and fALFF are caused by the different total power between the normal control and the TS patients. We plotted the values of ALFF, fALFF, and the total power in the significant clusters located by the ALFF or fALFF (Figure S2). For example, in Figure S2(a), both the ALFF (0.01–0.08 Hz) and the total power (0–0.25 Hz) were decreased in the left fusiform of the TS group, which, however, led the fALFF (ALFF divided by the total power) to remain almost unaltered in the TS group. Similar phenomena can be observed in Figure S2(b–d) that ALFF was changed while fALFF remained unchanged. While there is a slight difference between Figure S2(e) and the first four sub-figures (S2(a–d)) in that the former witnesses change in fALFF and no change in ALFF, the mechanism behind these 5 subfigures are the same in that the discrepancies between ALFF and fALFF lie in changes in the total power. These results demonstrate that the changes in the power in specific frequency band may be different from those in the total power for different groups. Although currently we do not know what causes the difference between them and what the potential physiological significance is, the results suggest that we should focus on the changes of the total power as well. Similar conclusions were also drawn in Zuo’s paper^53^ in that both ALFF and ALFF should be taken into consideration.

### Limitations

Firstly, the given sample size for the pure TS and the normal controls is relatively small. Secondly, TS patients are usually strongly comorbid with ADHD (about 50%) or OCD (20–60%)^54^. It is worthwhile to investigate the potential effects of comorbidity on the alterations in TS patients’ brain in the future.

## Conclusions

In conclusion, the present study adopted the ALFF/fALFF and ReHo approach on rs-fMRI data to investigate the alterations of spontaneous neural activity in the pure TS children. Abnormalities in TS children include the altered neural activity in the vision-related structures, the insula, and the cerebellum. We further found that the ReHo of the right cerebellum was positively correlated with TS duration. These results shed light on the underlying neurophysiological mechanisms reflected in the intrinsic brain activity and support the notion of immature brain development and functional maturation in TS children.

## Electronic supplementary material

Supplementary Materials
