# Supplementary material for: Altered Spontaneous Brain Activity in Children with Early Tourette Syndrome: a Resting-state fMRI Study
Source: Sci Rep. 2017 Jul 6;7:4808. doi: 10.1038/s41598-017-04148-z (PMC5500479; doi:10.1038/s41598-017-04148-z)
Supplement: Supplementary file 1 — Supplementary Materials [file 41598_2017_4148_MOESM1_ESM.docx]

# Altered Spontaneous Brain Activity in Children with Early Tourette Syndrome: a Resting-state fMRI Study

Yue Liu^1a^, Jieqiong Wang^2,3a^, Jishui Zhang^4^, Hongwei Wen^2,3^,Yue Zhang^1^, Huiying Kang^1^, Xu Wang^4^,Wenfeng Li^1^ , Huiguang He^2,3*^, Yun Peng^1,*^

^1^ Department of Radiology, Beijing Children’s Hospital, Capital Medical University

^2^ State Key Laboratory of Management and Control for Complex Systems, Institute of Automation, Chinese Academy of Sciences, Beijing, China

3 University of Chinese Academy of Sciences, Beijing, China

^4^ Department of Neurology, Beijing Children’s Hospital, Capital Medical University, Beijing, China

^a^ These authors contributed equally to this work

Correspondence to:

Yun Peng, Department of Radiology, Beijing Children’s Hospital, Capital Medical University.

No.56 Nanlishi Road, West District, Beijing, 100045, China.

E-mail: ppengyun@yahoo.com

Huiguang He, State Key Laboratory of Management and Control for Complex Systems,

Institute of Automation, Chinese Academy of Sciences, Beijing, 100190, China.

E-mail: [huiguang.he@ia.ac.cn](mailto:huiguang.he@ia.ac.cn)

**Supplementary Materials**

**Figures**


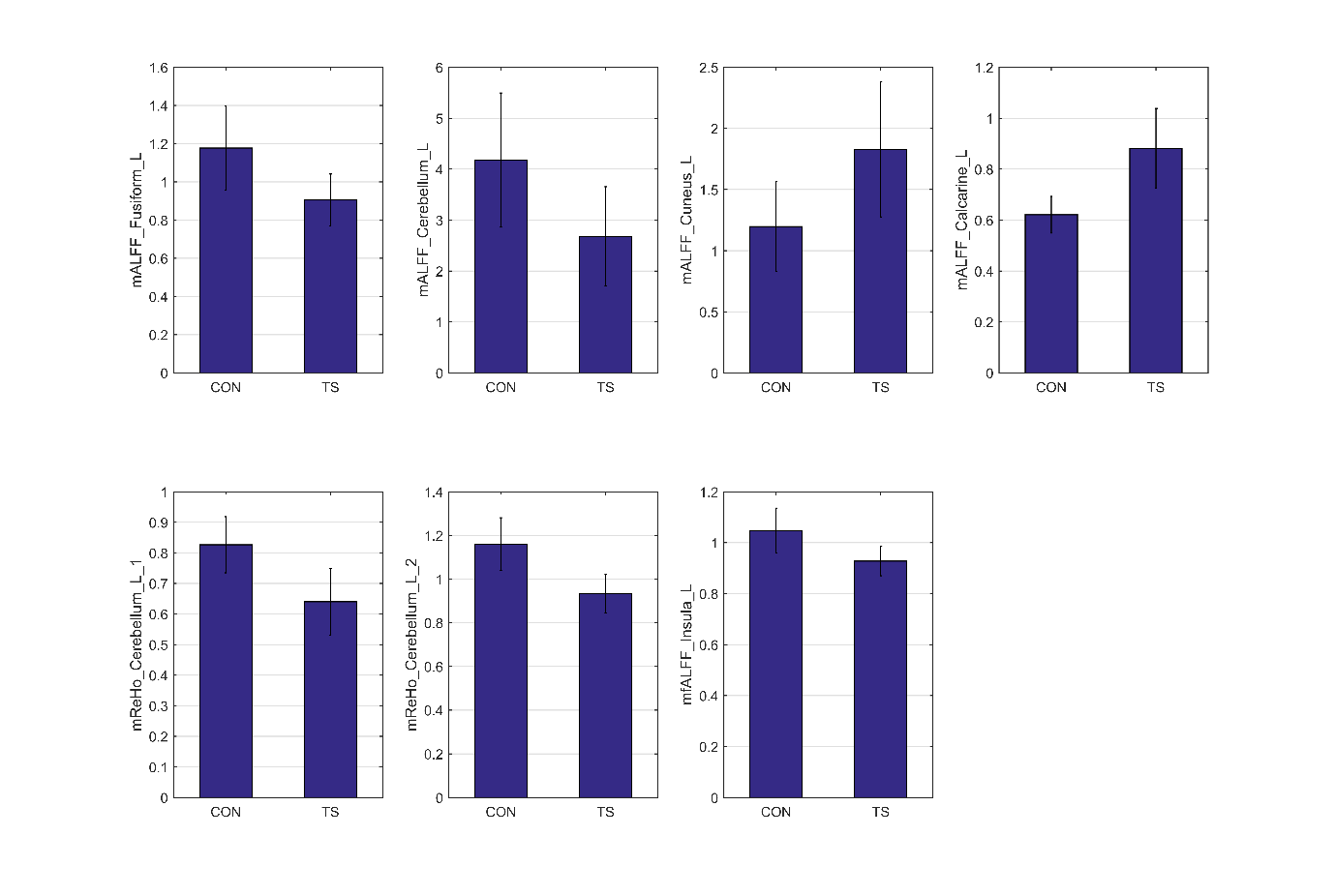


Figure S1 mALFF/mfALFF/mReHo values of ROIs that showed significant differences between normal controls and TS.


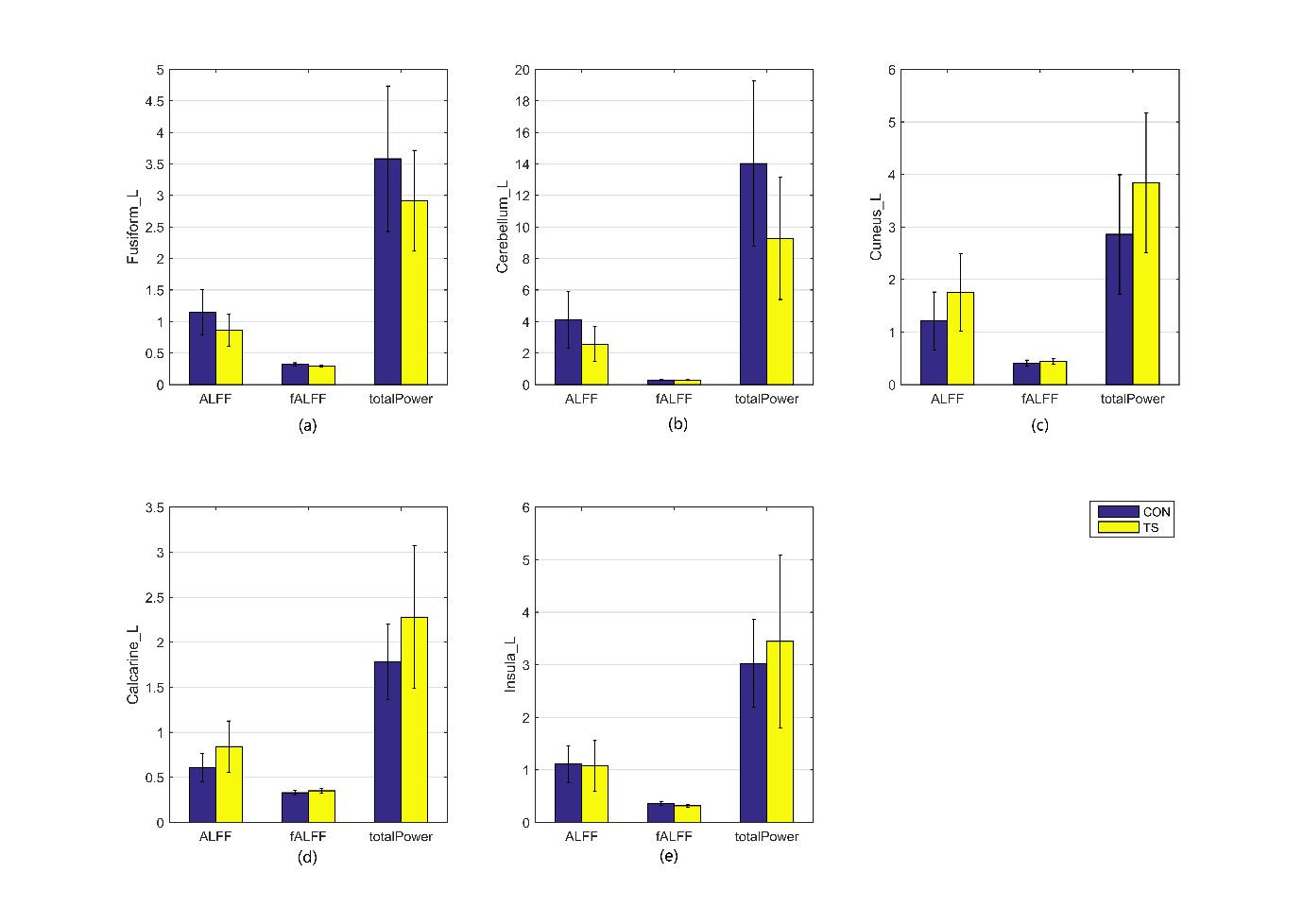


Figure S2 Values of ALFF/fALFF/total power in ROIs that showed significant differences between normal controls and TS via ALFF or fALFF.
